# Chemical Characterization and Leishmanicidal Activity In Vitro and In Silico of Natural Products Obtained from Leaves of *Vernonanthura brasiliana* (L.) H. Rob (Asteraceae)

**DOI:** 10.3390/metabo13020285

**Published:** 2023-02-16

**Authors:** Yuri Nascimento Fróes, João Guilherme Nantes Araújo, Joyce Resende dos Santos Gonçalves, Milena de Jesus Marinho Garcia de Oliveira, Gustavo Oliveira Everton, Victor Elias Mouchrek Filho, Maria Raimunda Chagas Silva, Luís Douglas Miranda Silva, Lucilene Amorim Silva, Lídio Gonçalves Lima Neto, Renata Mondêgo de Oliveira, Mylena Andréa Oliveira Torres, Luís Cláudio Nascimento da Silva, Alberto Jorge Oliveira Lopes, Amanda Silva dos Santos Aliança, Cláudia Quintino da Rocha, Joicy Cortez de Sá Sousa

**Affiliations:** 1 Microbial Pathogenicity Laboratory, CEUMA University, São Luís 65075-120, Brazil; 2 Laboratory of Research and Application of Essential Oils, Federal University of Maranhão, São Luís 65080-805, Brazil; 3 Laboratory of Environmental Sciences, CEUMA University, São Luís 65075-120, Brazil; 4 Immunophysiology Laboratory, Federal University of Maranhão, São Luís 65080-805, Brazil; 5 Virology Laboratory, CEUMA University, São Luís 65075-120, Brazil; 6 Central Laboratory of Public Health of the State of Maranhão, IOC-LACEN, São Luís 65020-320, Brazil; 7 Federal Institute of Science Education and Technology of Maranhão, São Luis 65300-000, Brazil; 8 Natural Products Research Laboratory, Department of Chemistry, Federal University of Maranhão, São Luís 65080-805, Brazil

**Keywords:** antileishmanial, medicinal plant, toxicity, *Vernonanthura brasiliana*

## Abstract

*Vernonanthura brasiliana* (L.) H. Rob is a medicinal plant used for the treatment of several infections. This study aimed to evaluate the antileishmanial activity of *V. brasiliana* leaves using in vitro and in silico approaches. The chemical composition of *V. brasiliana* leaf extract was determined through liquid chromatography-mass spectrometry (LC-MS). The inhibitory activity against *Leishmania amazonensis* promastigote was evaluated by the MTT method. In silico analysis was performed using Lanosterol 14alpha-demethylase (CYP51) as the target. The toxicity analysis was performed in RAW 264.7 cells and *Tenebrio molitor* larvae. LC-MS revealed the presence of 14 compounds in *V. brasiliana* crude extract, including flavonoids, flavones, sesquiterpene lactones, and quinic acids. Eriodictol (ΔGbind = −9.0), luteolin (ΔGbind = −8.7), and apigenin (ΔGbind = −8.6) obtained greater strength of molecular interaction with lanosterol demethylase in the molecular docking study. The hexane fraction of *V. brasiliana* showed the best leishmanicidal activity against *L. amazonensis* in vitro (IC_50_ 12.44 ± 0.875 µg·mL^−1^) and low cytotoxicity in RAW 264.7 cells (CC_50_ 314.89 µg·mL^−1^, SI = 25.30) and *T. molitor* larvae. However, the hexane fraction and Amphotericin-B had antagonistic interaction (FICI index ≥ 4.0). This study revealed that *V. brasiliana* and its metabolites are potential sources of lead compounds for drugs for leishmaniasis treatment.

## 1. Introduction

Leishmaniasis is among the neglected tropical diseases (NTDs). It has a wide geographic distribution and is endemic in underdeveloped countries [1,2]. According to the World Health Organization (WHO), an estimated 12 million people are infected annually, with about 20.000 to 30.000 deaths, and 350 million people are at risk of infection [2]. It is a complex and spectral anthropozoonosis caused by several species of the *Leishmania* genus. According to the infecting species and the immunological development of each person, it can evolve into different clinical forms. Among the clinical presentations of leishmaniasis, the cutaneous form stands out, which has *Leishmania amazonensis* as the major causative agent [3].

Pentavalent antimonials are still the drugs of choice for treating the disease. These medications have high toxicity, adverse effects during and after treatment, and prolonged time of parenteral administration, which leads to low patient adherence to treatment [4,5]. In addition, when used in sub-doses or discontinuously, they do not have the desired effect, which favors parasite resistance or relapses [6,7]. This context indicates the need to discover new alternatives for therapy against leishmaniasis. 

A wide range of traditional medicine and natural products can be considered for therapy against several diseases, including COVID-19 [8,9], cancer [10,11], parasitic infections [12,13], diabetes [14,15], and obesity [16]. In fact, the WHO recognizes the importance of traditional medicine, especially in underdeveloped countries, where approximately 85% of the population uses medicinal plants as a therapeutic alternative. In Brazil, the species *Vernonanthura brasiliana* (L.) H. Rob. belongs to the list of plants of interest to the public health system. *V. brasiliana*, popularly known as “assa-peixe”, is endemic to the Brazilian Cerrado (Brazilian Savannah) biome but can be found in other biomes due to its cosmopolitan and pantropical aspect [17,18,19,20]. It belongs to the *Asteraceae* family, the tribe *Vernonieae*, with more than 1000 species cataloged in the tribe. Among these, some species have been used in traditional medicine to treat numerous diseases [17].

The plants from the genus *Vernonanthura* are widely used, especially the leaves and roots, in cases of flu, colds, cough, bronchitis, bruises, hemorrhoids, rheumatism, hepatic colic, bleeding, and uterine infections [17,21]. Studies show the potential of this plant as a therapeutic alternative with anti-inflammatory, antimicrobial, antifungal, insecticidal, antioxidant, immunomodulatory, anthelmintic, antinociceptive, and antiprotozoal action [12,18,22,23,24,25]. Some studies also report the activity of the essential oil of *V. brasiliana* against *L. amazonensis*, *L. infantum*, and *L. donovani* promastigotes [23,26,27].

Due to the difficulties in treating leishmaniasis, this research aimed to investigate the anti-*Leishmania* action of *V. brasiliana* extract and fractions. The in silico interactions of the compounds detected in the *V. brasiliana* extract were assessed using the enzyme cytochrome P450 Lanosterol 14alpha-demethylase (CYP51) as the target. Furthermore, the toxicity of these products was evaluated in the RAW 264.7 cell line and *Tenebrio molitor* larvae, seeking to support the idea of this plant as a source for new compounds with less toxicity and leishmanicidal efficacy.

## 2. Materials and Methods

### 2.1. Plant Material

Aerial parts of *V. brasiliana* were collected in the city of Bacabeira, Maranhão State, Brazil (3°03′34.5″ S 44°20′12.4″ W), at the dry period, 8:00 a.m. The plant material was identified at the Herbarium “Prisco Bezerra” of the Federal University of Ceará, and a voucher specimen was deposited (number 55227). 

### 2.2. Preparation of Crude Extracts and Fractions

The leaves were dried and ground in a knife mill. The hydroalcoholic crude extract of *V. brasiliana* (EB*Vb*) was obtained after maceration and agitation in 70% ethyl alcohol for seven days, in a proportion of 3:1 (v:m) (alcohol and plant material, respectively). The aqueous crude extract was obtained in the same conditions as EB*Vb* [28]. EB*Vb* was concentrated in a rotary evaporator (Fisatom^®^ 802, São Paulo, SP, Brazil) and lyophilized (Terroni^®^ Enterprise, São Carlos, SP, Brazil). EB*Vb* (1 g) was successively partitioned with hexane, ethyl acetate, and methanol to obtain the respective fractions (FH*Vb*, FAE*Vb*, and FM*Vb*). The samples were stored under refrigeration and protected from light until analysis.

### 2.3. Liquid Chromatography-Mass Spectrometry Analyses (LC-MS)

For chemical characterization, 10 mg of each sample was dissolved in methanol (1.0 mL). The samples were filtered on a PTFE^®^ filter (0.22 µm and 20 mL) and injected into a liquid chromatograph coupled to a mass spectrometer (Amazon Speed^®^ ETD-Bruker, Billerica, MA, USA). The chromatographic profile of EB*Vb* and fractions were analyzed in the LCQ mass spectrometer (Thermo Scientific^®^, San Jose, CA, USA), equipped with a column (Phenomenex^®^ Luna C18 columns, California, CA, USA) 250 mm × 4.6 mm; 5 μm, with a flow rate of 0.9 mL/min. The identification of EB*Vb* compounds and fractions was performed by fragmentation mechanisms in negative mode, comparing mass spectral data with the literature.

### 2.4. In Silico Studies and Molecular Docking

The compounds identified in EB*Vb* were structurally schematized in 3D with the GaussView^®^ 5.0.8, Wallingford, CT, EUA, program [29]. The geometric and vibrational properties were calculated and vacuum optimized at the Density Functional Theory (DFT) level, using the hybrid function B3LYP combined with the base set 6–31 ++ G (d, p) with the Gaussian^®^ 09, Wallingford, CT, EUA [30]. 

The sterol 14-alpha-demethylase (CYP51) of *L. infantum* (99.3% similarity with *L. amazonensis*) was obtained from the Protein Data Bank (PDB) (#3L4D), resolved by X-ray crystallography with a resolution of 2.75 Å. Fluconazole and other molecules in the crystal were removed, keeping only one of the two homologous chains and the HEME group. 

Molecular docking procedures were performed using Autodock^®^ Vina (La Jolla, California, EUA) [31]. The *L. infantum* CYP51 (LiCYP51) structure and ligands were prepared for molecular anchorage calculations using Autodock^®^ Tools 1.5.7, (La Jolla, California, EUA [32]. The docking methodology described in the literature [33] was used, with modifications [33]. Gasteiger partial charges were calculated after adding all hydrogens for both ligands and LiCYP51 structure. The dimensions of the cubic box on the X, Y, and Z axes were 30 × 30 × 30. The grid box was centered on the Iron atom of the LiCYP51 HEME group.

The conformations of the initial interaction coordinates of the LiCYP51 complexes and *V. brasiliana* compounds were chosen based on the best binding free energy parameters and visual inspection.

### 2.5. Parasites

Promastigote forms of *L. amazonensis* (MHOM/BR/1987/BA-125) were cultured at 26 °C in Schneider’s Insect Medium, supplemented with 10% fetal bovine serum, 100 U/mL of penicillin, and 100 μg·mL^−1^ of streptomycin.

### 2.6. Activity against Promastigote Forms

*L. amazonensis* promastigotes (10^6^ parasites. mL^−1^) were plated into 96-well plates and treated with different concentrations of EB*Vb* and fractions obtained by serial dilutions (512 to 4 µg·mL^−1^). After 24, 48, and 72 h of incubation, the viability of the parasites was measured by the colorimetric method with tetrazolium-dye 3-(4,5-dimethylthiazol-2-yl)-2,5-diphenyltetrazolium bromide (MTT) [34] and Neubauer chamber counting. Aspects such as mobility, size, and shape of the parasites were also evaluated. MTT solution (10 μL; 5 mg·mL^−1^) was added to each well and, after four hours, 100 μL of DMSO was added to dissolve the formazan crystals. The absorbance was analyzed on a spectrophotometer at a wavelength of 570 nm. Data were normalized using the formula: % survival = sample OD − blank OD/control OD − blank OD × 100. The results were used to calculate the IC_50_ (inhibitory concentration for 50% of parasites). Meglumine antimoniate and amphotericin-B were used as reference drugs. All tests were performed in triplicate and repeated at least twice.

### 2.7. Fluorescence Microscopy

*L. amazonensis* promastigotes (10^6^ parasites·mL^−1^) were incubated with EB*Vb* and FH*Vb* (1 × IC_50_ and 2 × IC_50_). After 48 h, acridine orange (3,6-dimethyl-amino-acridine; 10 µg·mL^−1^) was added to each sample, for 20 min. Phosphate buffer solution, 70% methanol, and Amphotericin-B were used as controls. For fluorescence evaluation, slides were prepared and analyzed in Axio Imager fluorescence microscope (Zeiss^®^, Jena, Germany) (Alexa Filter-488 nm).

### 2.8. Determination of Drug Interactions

After preliminary tests, the hexane fraction of *V. brasiliana* (FH*Vb*) and amphotericin-B were selected to conduct drug interaction assays. The interaction of the two substances was assessed using the adapted isobologram method [35]. The IC_50_ values of the compounds were used to establish the concentrations of each drug in the combination:

Combination 1 (5:0): 160 µg·mL^−1^ FH*Vb* + 0.0 µg·mL^−1^ amphotericin-B.

Combination 2 (4:1): 80 µg·mL^−1^ FH*Vb* + 0.25 µg·mL^−1^ amphotericin-B.

Combination 3 (3:2): 40 µg·mL^−1^ FH*Vb* + 0.5 µg·mL^−1^ amphotericin-B.

Combination 4 (2:3): 20 µg·mL^−1^ FH*Vb* + 1 µg·mL^−1^ amphotericin-B.

Combination 5 (1:4): 10 µg·mL^−1^ FH*Vb* + 2 µg·mL^−1^ amphotericin-B.

Combination 6 (0:5): 5 µg·mL^−1^ FH*Vb* + 4 µg·mL^−1^ amphotericin-B.

The experiments were performed in the same way as described in item 2.6. After 72 h, the viability of *L. amazonensis* promastigotes were measured by Neubauer chamber counting. The results were normalized in percentage. After data normalization, fractional inhibitory concentrations (FIC) at the IC_50_ level were calculated for both drugs. The value obtained was used to classify the nature of the interaction as synergistic (FICI < 0.5), additive (0.5 < FICI < 4), or antagonist (FICI > 4).

### 2.9. Cytotoxicity Assay in RAW 264.7 Cells

The RAW 264.7 cell line, provided by the Immunophysiology Laboratory of the Federal University of Maranhão—UFMA, was cultured in RPMI medium, supplemented with 10% FBS, 20 mM of L-glutamine, 7.5% sodium bicarbonate, penicillin (100 μg·mL^−1^), and streptomycin (50 μg·mL^−1^), at 37 °C and 5% CO_2_. The cytotoxic effect of natural products of *V. brasiliana* was performed using the RAW 264.7 cell line. The cells were seeded in 96-well plates (5 × 10^4^ cells/well) and, after 24 h, were treated with different concentrations of EB*Vb* and FH*Vb* (1024 to 8 µg·mL^−1^). After 48 h, cell viability was measured by MTT colorimetric assay [34,36]. Culture medium and DMSO 20% (40 µg·mL^−1^) were used, respectively, as negative and cytotoxic controls. The plates were analyzed in a microplate reader, at a wavelength of 570 nm. Data were normalized for CC_50_ (cytotoxic concentration for 50% of cells) calculation and the selectivity index (CC_50_/IC_50_ ratio). Tests were performed in triplicate.

### 2.10. Toxicity in Tenebrio Molitor Larvae

In vivo toxic effect of the compounds with leishmanicidal activity was evaluated on larvae of the insect *T. molitor*. Larvae (100.0 mg) were randomly divided into groups (10 larvae/group). Before inoculating the natural compounds, the cuticles were cleaned with 70% alcohol, and then 10.0 µL of each test solution was injected. Glucantime^®^ was used as a positive control and 1% PBS as a negative control. The survival curve was determined by the absence of movement or total melanization of the larvae over five days. The damage caused by natural products used in *T. molitor* larvae was also evaluated. The degree of suffering was observed through melanization, movement, reaction to stimuli, and survival [37].

### 2.11. Statistical Analyses

Values were expressed as mean ± standard deviation. The results were analyzed using a two-way analysis of variance (ANOVA) followed by the Tukey post hoc test. For the toxicity tests in *T. molitor* larvae, the log-rank test (Mantel–Cox) was performed, and to evaluate the difference between the severity degree, the two-way ANOVA was used. Differences were considered significant when *p* < 0.05.

## 3. Results

### 3.1. Chromatography and Identification of Compounds

**Figure 1:**
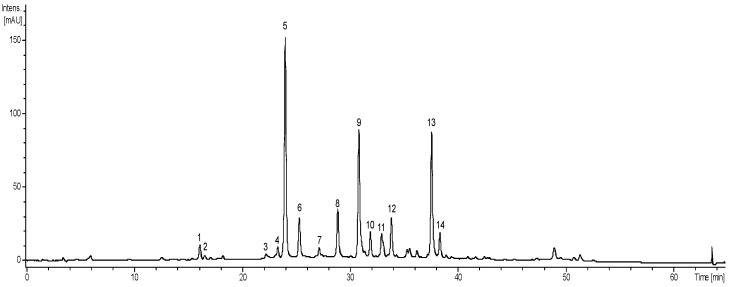
Total ion chromatogram generated by LC-EM (270 nm) of the Hydroalcoholic Crude Extract of *Vernonanthura brasiliana* (EB*Vb*).

To chemically characterize and prospect the studied species, correlating it with its biological potential investigated in this study, the EB*Vb* was submitted to chromatographic analysis (LC-MS), and 14 peaks were identified, listed according to the retention time, as shown in Figure 1.

The identified compounds are classified as flavonoids, flavones, sesquiterpene lactones, and quinic acids. The identification was elucidated by comparing data obtained by LC-MS with the fragmentation profiles described in the literature (Table 1).

### 3.2. In Silico Studies and Molecular Docking

For molecular docking calculations, all compounds identified in EB*Vb* were evaluated. Among the evaluated compounds, the best free binding energy parameters were presented by eriodyctiol, luteolin, and apigenin, with values of −9.0 kcal/mol, −8.7 kcal/mol, and −8.6 kcal/mol, respectively. 

In addition to the compounds present in the extract, the azole antifungal fluconazole was also anchored. It is observed that eriodyctiol, luteolin, and apigenin showed higher affinity parameters than the antifungal (Table 2). Fluconazole is the native molecule of the LiCYP51 (*Leishmania infantum* CYP51) crystal structure, so fluconazole redocking was performed to validate the docking protocol. The root-mean-square deviation (RMSD) between the predicted coupling conformation and the observed X-ray crystal structure was 1.47 Å. Values below 2 Å indicate that the coupling protocol is valid. Other compounds identified in the *V. brasiliana* extract showed more discrete affinity parameters when compared with eriodyctiol, luteolin, and apigenin, but still suggested a favorable interaction with LiCYP51. The results of the free binding energy values of all ligands are shown in Table 2.

**Figure 2:**
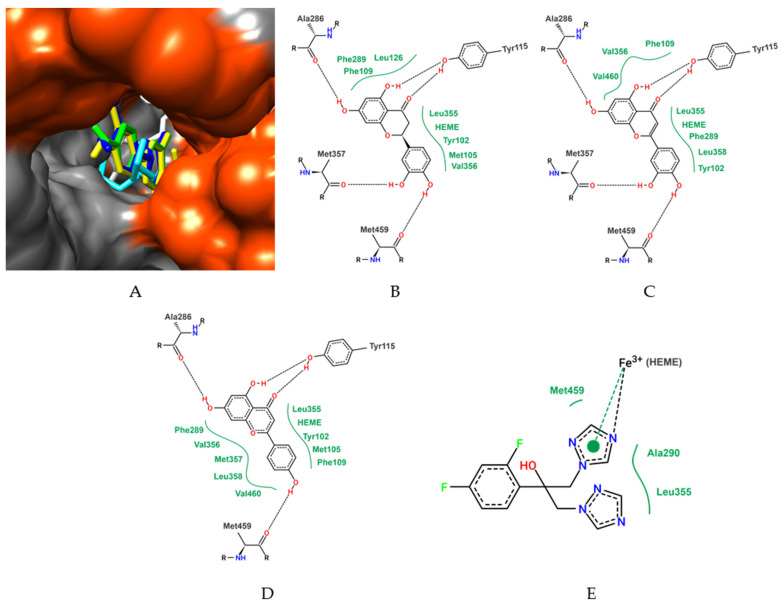
Detailed representation of conformations obtained by molecular docking of eriodictyol (blue), luteolin (yellow), apigenin (green), fluconazole (cyan), and HEME group (white) at the LiCYP51 active site (**A**). Two-dimensional diagram of the interactions performed by eriodyctiol (**B**), luteolin (**C**), apigenin (**D**) and fluconazole (**E**) with the amino acid residues and HEME group of the LiCYP51 active site. Dashed black lines represent hydrogen bonds, while full green lines represent van der Waals interactions.

Evaluating the LiCYP51 complex with the ligands and the best binding free energy parameters, it is observed that all ligands performed stable interactions with amino acid residues of the active site of LiCYP51, those being eriodictyol with hydrogen bonds with Tyr115, Ala286, Met357 and Met459 residues, and van der Waals interactions with Tyr102, Met105, Phe109, Leu126, Phe289, Val356 residues and with the HEME group (Figure 2).

Luteolin was stabilized with the amino acid residues of LiCYP51 by hydrogen bonds with the residues Tyr115, Ala286, Met357, and Met459, and van der Waals interactions with Tyr102, Phe109, Phe289, Val356, Leu358, and Val460 residues and with the HEME group. Another molecule is apigenin with stabilization in Tyr115, Ala286, Met459 residues (hydrogen bonds) and Tyr102, Met105, Phe109, Phe289, Val356, Met357, Leu358 and Val460, and the HEME group (van der Waals) (Figure 2).

### 3.3. Leishmanicidal Activity

The natural products from the leaves of *V. brasiliana* demonstrated action against the promastigote forms of *L. amazonensis*, with satisfactory in vitro inhibition, characterized by low IC_50_ values, at the three evaluated times, 24 h, 48 h, and 72 h. The lowest IC_50_ was for FH*Vb*, equal to 5.76 µg·mL^−1^ in the first 24 h of treatment, as shown in Table 3.

EB*Vb* showed a moderate concentration-dependent inhibition against the promastigote forms between 24 and 72 h of exposure. There was no significant difference between the times of 24 and 48 h (*p* = 0.8890); however, there was a difference between the times of 48 and 72 h due to the reduction of the IC_50_ (*p* < 0.0001).

FAE*Vb* also showed moderate activity against the parasites, but there was a resumption of parasite growth and an increase in IC_50_ between 24 and 72 h (*p* < 0.0001). FH*Vb* showed a significant difference between its IC_50_ values in the incubation times (*p* < 0.0001). The FM*Vb* showed very divergent IC_50_ between the times evaluated, suggesting instability of the fraction.

Among the fractions of *V. brasiliana*, FH*Vb* was the one that best induced parasitic inhibition at 24 and 48 h of exposure, showing a significant difference between its IC_50_ values in the treatment times (*p* < 0.0001).

The first-choice reference drug, meglumine antimoniate, was ineffective against the clinical isolate used in our in vitro assays. Little or no change was observed in the mobility and size of the parasites during 48 h of exposure. Therefore, it was not used as a positive control for the experiment. However, amphotericin-B, used as a chemotherapy drug of second choice in Brazil, proven to be effective, destroying or reducing the size and mobility of the parasites at very low concentrations. 

**Figure 3:**
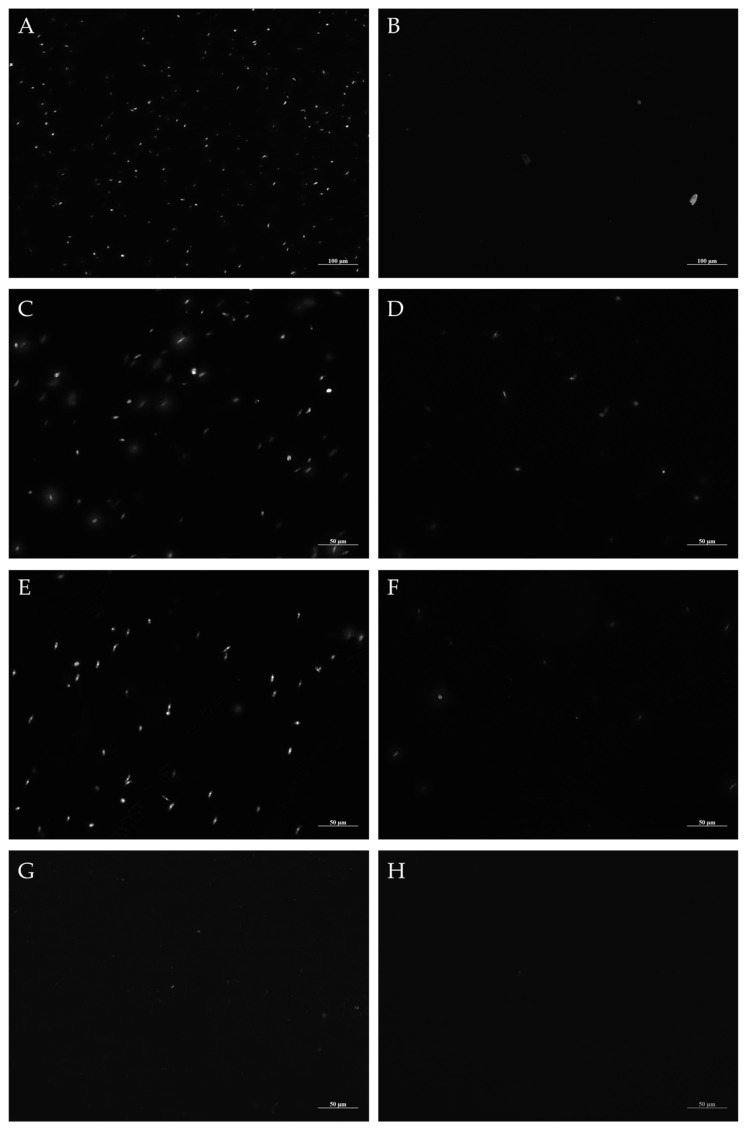
Microscopic fluorescence analysis of promastigote forms of *Leishmania amazonensis* after treatment for 48 h with natural products of *Vernonanthura brasiliana* (L.) H. Rob. Viable parasites were labeled with acridine orange and presented in dark field. (**A**) Parasites in *Schneider medium*; (**B**) Parasites with 70% methanol; (**C**) Parasites with EB*Vb*-IC_50_; (**D**) Parasites with EB*Vb*-2xIC_50_; (**E**) Parasites with FH*Vb*-IC_50_; (**F**) Parasites with FH*Vb*-2xIC_50_; (**G**) Parasites with Amphotericin-B-IC_50_; (**H**) Parasites with amphotericin-B-2xIC_50_. To capture the images, the Alexa filter (488 nm) was used in the Zeiss^®^ Axio Fluorescence Microscope Imager (50 μm and 100 μm).

To verify and illustrate the action of the natural products evaluated on the promastigote forms of *L. amazonensis*, an analysis was carried out by fluorescence microscopy, using acridine orange. The exposure for 48 h of the promastigote forms to the IC_50_ of the test solutions, EB*Vb*, FH*Vb*, and amphotericin-B, confirmed the leishmanicidal action of the tested products, with a reduction of more than half of the load of the parasitic inoculum, which was standardized at a concentration of 1.10^6^ mL^−1^ cells. Also noteworthy is a flagellar shortening in the promastigote forms of *L. amazonensis* treated with FH*Vb* and amphotericin-B (Figure 3).

Furthermore, when using concentrations of twice the IC_50_ value, there was an almost total reduction of the parasites, in agreement with the indices of leishmanicidal activity obtained. The controls, negative (only parasites in Schneider culture medium) and positive (parasites treated with 70% methanol) were as expected, agglomeration of promastigote forms and destruction of promastigote forms, respectively (Figure 3).

### 3.4. Determination of Drug Interactions

Among the natural products evaluated, FH*Vb* presented the best results, and so was chosen for the drug interaction test. The results of the interaction analysis, combined or isolated in each association, are shown in Table 4. The interaction between FH*Vb* and amphotericin-B was classified as antagonistic, with a FICI index greater than or equal to 4.0.

In the first combination, no *L. amazonensis* promastigotes were seen, therefore, there was a clear field of view. In the second combination, only one promastigote form was seen per field of view, with low mobility and flagellar shortening. The other combinations and amphotericin-B alone reduced the cell viability of the parasites by 100%. These results classified FH*Vb* as a natural product with antagonistic action (FICI ≥ 4.0) on amphotericin-B in vitro.

### 3.5. In Vitro Cytotoxicity

In the search for a natural product with less toxicity and leishmanicidal efficiency, cytotoxic tests were conducted in RAW 264.7 cells for the natural compounds of *V. brasiliana*, with better leishmanicidal action. After 48 h of exposition, the cytotoxic concentration values for 50% of the cells (CC_50_) ranged from 8 to 314, 8 µg·mL^−1^ (Table 5).

EB*Vb* and FAE*Vb* showed low CC_50_ values. The EB*Vb* showed abnormal data, and the lowest concentration used for the assays (8 µg·mL^−1^) resulted in a total reduction of cell viability in 48 h, a result confirmed with visualization under an inverted optical microscope and a Neubauer chamber.

FAE*Vb* showed a moderate value of CC_50_; however, when the ratio between its indices (CC_50_/IC_50_) was calculated as a low index, selectivity was obtained. FH*Vb* was the least cytotoxic among the evaluated compounds, presenting high selectivity to the parasites (SI = 25.3). The cytotoxic control (DMSO) reduced the cell viability by 100% in 48 h of exposure; therefore, the confidence interval values and data correlation (R²) were not determined.

### 3.6. In Vivo Toxicity against Tenebrio Molitor

**Figure 4:**
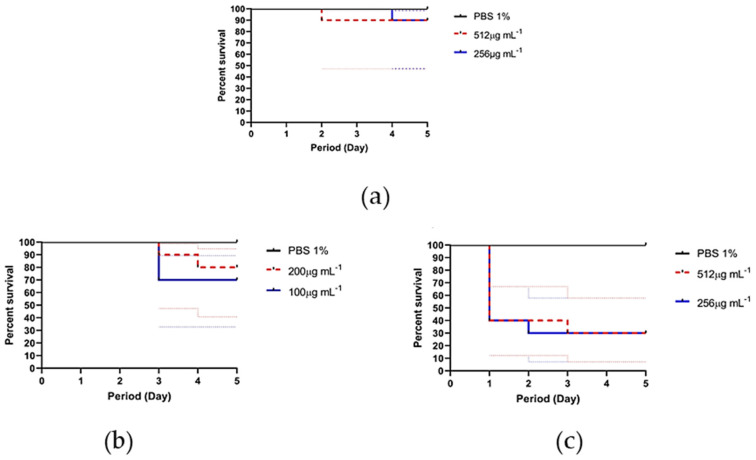
Survival curve of *Tenebrio molitor* larvae treated with crude extract or hexane fraction of *Vernonanthura brasiliana* (L.) H. Rob and leishmanicidal reference drug Glucantime^®^. The larvae were treated and evaluated daily for a period of 5 days. (**a**) Larvae with hydroalcoholic crude extract (EB*Vb*); (**b**) Larvae with Glucantime^®^; (**c**) Larvae with hexane fraction (FH*Vb*). Solid lines represent survival over time and dotted lines represent the 95% confidence interval. Larvae treated with sterile 1% PBS were considered negative control.

Subsequently, it was decided to conduct an assay of the natural products, EB*Vb* and FH*Vb*, and the leishmanicidal reference drug (Glucantime^®^), in an alternative model (in vivo), with larvae of *T. molitor.* The larvae exposed to Glucantime^®^ suffered melanization and reduced mobility, an aspect of suffering from the third day of evaluation, where 30% of the larvae did not resist a concentration of 200 µg·mL^−1^. This fact was noticed with greater intensity for the EB*Vb*: in 24 h of exposition, only 40% of the larvae were alive, also with melanization and low mobility. After the third day, only 30% of the larvae were alive at both concentrations used in the assays (Figure 4).

The percentage of larval survival against exposure to FH*Vb* was high compared to the others evaluated, where only one larva did not survive in both concentrations evaluated, that is, 90% of the larvae survived. The larvae that remained alive until the fifth day did not show melanization, and remained with unaltered mobility (Figure 4).

**Figure 5:**
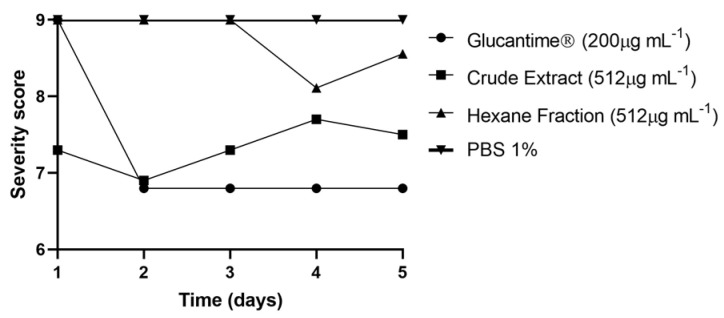
Evaluation of severity score of *Tenebrio molitor* larvae treated with crude extract or hexane fraction of *Vernonanthura brasiliana* (L.) H. Rob and leishmanicidal reference drug Glucantime^®^. The difference between the tested groups was considered significant when *p* < 0.05 using two-way ANOVA.

When multiple comparisons of the degree (score) of the suffering of larvae exposed to natural products (EB*Vb* and FH*Vb*) and Glucantime^®^ were performed, there was no difference between EB*Vb* and Glucantime^®^ (*p* = 0.9958) and the score was lower. However, FH*Vb* was the one that least attacked the larvae (highest score), with no difference from the negative control (PBS 1%) (*p* = 0.9344)(Figure 5).

## 4. Discussion

This research evaluated the chemical composition of natural products obtained from the leaves of *V. brasiliana*, to elucidate their ability to inhibit the growth of promastigote forms of *L. amazonensis*, in addition to evaluating the in vitro cytotoxicity and in vivo toxicity, an alternative model.

The genus *Vernonanthura* has bioactive potential as they are rich in terpenes and sesquiterpenes, with reports of in vitro antiplasmodic, anti-*Leishmania*, antimicrobial, anti-schistosomiasis, and anti-inflammatory activity [20]. This genus has previous reports of activity against the promastigote forms of *L. amazonensis* and *L. infantum* species [12,28].

Chemical and chromatographic analyses of EB*Vb* revealed the presence of 14 compounds, classified as flavonoids, flavones, sesquiterpene lactones, and quinic acids. There was disagreement regarding the number and types of compounds identified in our research group by previous chromatographic analyses, where they identified 24 different compounds in the same plant species. It is understood that the variation of metabolites produced by a plant can be attributed to physical, chemical, and biological factors (phytopathogens) and edaphoclimatic characteristics (weather, climate, wind, altitude, etc.) [38,39]. 

In silico studies were conducted to evaluate the binding strength between the compounds identified in this work, described in Table 1. The flavonoids eriodyctiol, luteolin, and apigenin obtained higher binding energy with Lanosterol demethylase [40], a fundamental compound for the biosynthesis of ergosterol specific to the *Leishmania* genus. Lanosterol demethylase is an enzyme complex (P45014DM) essential for ergosterol metabolic pathways that participate in the organization of the cytoplasmic membrane of the parasites [41].

Amphotericin-B is a drug that traditionally acts on fungal infections, binding to ergosterol in the plasma membranes of the cells of these organisms, causing disruption of membrane function, and allowing the leakage of electrolytes (particularly potassium) and small molecules, resulting in death of the cell [42], and has been successfully used for the treatment of Leishmaniasis. Another noteworthy fungicide fluconazole, which inhibits CYP51, which is responsible for the synthesis of ergosterol; this drug is also capable of inhibiting CYP51 of *L. infantum* [43]. Thus, CYP51 is a relevant target for research of compounds with leishmanicidal activity. This enzyme plays a key role in the synthesis of lanosterol to ergosterol. Ergosterol is an essential compound in the fungal cell membrane, and once lanosterol accumulation occurs due to non-conversion to ergosterol, the plasma membrane is disrupted, damaging the cell [42,43].

Based on the results obtained, it was observed that EB*Vb* and its fractions have leishmanicidal activity against *L. amazonensis*. The compounds identified in the extract were submitted to molecular docking to evaluate the possible interaction of these compounds with the crystallographic structure of LiCYP51, which has a similarity of 99.3% with the enzyme of *L. amazonensis*, which was not available. Negative values of free binding energies indicate that these interactions are favorable for the formation of the ligand-receptor complex [33]. Through molecular docking, we found that, among the compounds identified in the extract, eriodictiol, luteolin, and apigenin were the compounds that presented the most favorable parameters for complex formation with LiCYP51. 

Thus, our results suggest that the biological activity of *V. brasiliana* against *L. amazonensis* may also be associated with an inhibition of ergosterol biosynthesis, mediated by CYP51. Thus, it is inferred that the metabolites of *V. brasiliana* can be considered potential new leishmanicidal candidates. These metabolites found in *V. brasiliana* have already been detected in other plants, such as *Pistacia atlantica* Desf and *Limonium aureum*, species found in the East. *P. atlantica* has been reported to reduce the development of cutaneous lesions triggered by *Leishmania major* in Balb/c [44,45,46]. 

Some molecules present in EB*Vb* have antimicrobial and leishmanicidal activities, such as eriodictiol, previously isolated from *Psorothamnus polydenius* and *Limonium brasiliense*. Eriodictiol showed excellent inhibition against promastigote forms of *L. donovani* (IC_50_ = 25 ± 4 µg·mL^−1^, CC_50_ = 32.8 ± 12.3 µg·mL^−1^) with a reduction in the number of infected macrophages by 55 ± 16% [41,47], and against *Leishmania amazonensis* (IC_50_ = 12.38 µg·mL^−1^) [46,48]. Another example is the flavonoid luteolin which has in vitro and in vivo ability to inhibit *L. donovani*. In silico studies also revealed that luteolin can inhibit the action of topoisomerase II, an important kinetoplast DNA (kDNA) replication enzyme, promoting apoptosis of parasites and inducing low toxicity to mammalian cells [49,50,51]. 

Apigenin is another flavonoid with several known pharmacological actions, including antibacterial, anti-inflammatory, antioxidant, and leishmanicidal effects [52,53,54,55,56,57]. Apigenin inhibits arginase in *L. amazonensis* and *L. donovani* in vitro and in vivo; blocking this enzyme can trigger oxidative stress, controlling the infection. The effect of apigenin on *L. amazonensis* and *L. tropica* may be associated with the production of reactive oxygen species (ROS) leading to mitochondrial collapse [58,59,60]. Drug combinations, such as miltefosine and apigenin, have already been studied, demonstrating a reduction in the parasite load of the amastigote form of *L. amazonensis* in Balb/c mice [61,62].

In vitro studies about the interaction (synergistic, indifferent, or antagonistic effects) of natural products and reference drugs are few reported. An additive effect between Glucantime^®^ and a natural compound isolated against *L. amazonensis* has already been observed by Gonçalves-Oliveira et al. [62]. The synergism of isolated products can be effective for the development of new chemotherapeutics combined with natural compounds [62,63]. Moreover, the incorporation of natural products and conventional drugs into nanoformulations opens a new avenue for antileishmanial therapy [64,65,66].

The toxicity of the natural products used in this study was also evaluated using larvae of *T. molitor*. As expected, the reference drug for the treatment of cutaneous leishmaniasis (Glucantime^®^) presented high toxicity and suffering for *T. molitor* larvae compared to the negative control. When the larvae were exposed to EB*Vb*, suffering and a lower percentage of survival were also observed compared to FH*Vb*, which demonstrated the lowest toxicity, a low index of suffering, and a higher percentage of survival of *T. molitor* larvae.

## 5. Conclusions

The hexane fraction of *V. brasiliana* is a promising source of leishmanicidal compounds, demonstrating greater potency (dose inhibition) against parasites. This fraction has low toxicity towards RAW 264.7 cells and *T. molitor* larvae and showed a good selectivity index. The compounds identified in the crude extract, such as eriodictiol, luteolin, and apigenin, demonstrate excellent strength of molecular interaction on lanosterol demethylase, an important enzyme that participates in the formation of membrane ergosterol in the parasites. Therefore, these results reinforce the continuity of studies with this plant species for further biomedical explanations, further strengthening the idea of the hexane fraction as a raw material for the isolation of metabolites and/or development of new chemotherapeutics for the treatment of leishmaniasis.

## Figures and Tables

**Table 1:** Identification of compounds in the hydroalcoholic crude extract of *Vernonanthura brasiliana* by LC-ESI-IT-MS/MS in negative mode.

No.	Retention Time (min)	[M-H] ^-^	MS ^n^ Ion m/z (-)	Proposed Substance
1	16.2	353; 191	353	chlorogenic acid or 5-o-caffeoylquinic acid
2	16.7	353; 289; 203	515	3,5-dicapheoylquinic acid
3	22.2	287; 151	463	eriodictyol-7-o-glucuronide
4	23.4	375; 191	537	hydroxylariciresinol-hexoside
5	24.0	149	177	esculetin
6	25.4	285	923/461	luteolin-7-o-glucuronide
7	27.2	151	575/287	eriodictyol
8	28.9	275; 231	293	octadecadienoic acid
9	30.9	285	571	luteolin
10	32.9	269; 151	539/269	apigenin
11	33.1	284	299	hispidulin
12	35.5	299	599	chrysoeriol
13	37.7	298; 283	313	3’,4’-dimethoxy luteolin
14	41.6	171	293	gingerol

Retention time (minutes); [M-H]-: ionization in negative mode.

**Table 2:** Values of the free energy binding parameters of the compounds identified in the *Vernonanthura brasiliana* (L.) H. Rob extract with the *Leishmania infantum* CYP51.

*Leishmania infantum* CYP51	
Ligand	ΔGbind (kcal/mol)
eriodictyol	−9.0
luteolin	−8.7
apigenin	−8.6
hispidulin	−8.5
chlorogenic acid	−8.1
chrysoeriol	−8.0
3’,4’-dimethoxyluteolin	−8.0
gingerol	−7.3
luteolin-7-o-glucuronide	−7.2
eriodictiol-7-o-glucuronide	−7.1
esculetin	−6.9
quinic acid	−6.9
hydroxylariciresinol-hexoside	−6.8
Fluconazole	−8.1

**Table 3:** Leishmanicidal activity of the crude extract and fractions of *Vernonanthura brasiliana* (L.) H. Rob against the promastigote forms of *Leishmania amazonensis* at three treatment times.

Natural Products	24 h		48 h		72 h	
	IC_50_ ± SD (µg·mL^−1^)	R²	IC_50_ ± SD (µg·mL^−1^)	R²	IC_50_ ± SD (µg·mL^−1^)	R²
EB*Vb*	24.63 ± 0.375 ^a,1^	0.970	24.31 ± 0.625 ^a,1^	0.904	19.04 ± 0.750 ^b,1^	0.880
FH*Vb*	5.76 ± 0.250 ^a,2^	0.972	12.44 ± 0.875 ^b,2^	0.941	22.53 ± 0.125 ^c,2^	0.876
FAE*Vb*	21.78 ± 0.500 ^a,3^	0.947	28.24 ± 0.750 ^b,3^	0.953	54.89 ± 0.375 ^c,3^	0.879
FM*Vb*	60.63 ± 0.250 ^a,4^	0.940	353.3 ± 1.00 ^b,4^	0.948	60.13 ± 0.125 ^c,4^	0.862
Glucantime^®^	>500	ND	>500	ND	>500	ND
Amphotericin-B	0.1644 ± 0.500 ^a^	0.996	0.5388 ± 0.250 ^a^	0.991	0.7793 ± 0.375 ^a^	0.991

IC_50_—50% inhibitory concentration of promastigotes; ± Standard deviation; ND—Not determined. R²—Data correlation index; EB*Vb* (crude extract); FH*Vb* (hexane fraction); FAE*Vb* (ethyl acetate fraction); FM*Vb* (methanolic fraction); The comparison between the independent groups was performed by the ANOVA test (two-way) with the Tukey post-test. In each line, different superscript letters (^a–c^) indicate significant difference (*p* < 0.05). In each column, different superscript numbers (^1–4^) indicate significant difference (*p* < 0.05).

**Table 4:** Assessment of drug interaction between the hexane fraction of *Vernonanthura brasiliana* (L.) H. Rob with amphotericin-B against *Leishmania amazonensis* in the promastigote form.

Combination Ratio		Combined Drugs					FICI
		IC_50_ µg·mL^−1^		FIC_50_		∑FIC_50_	
FH*Vb*	Anf-B	FH*Vb*	Anf-B	FH*Vb*	Anf-B		
5	0	160	0	7.101	0	7.101	23,691
4	1	80	0.25	3550	0.320	3.870	
3	2	40	0.5	1775	0.641	2416	
2	3	20	1	0.887	1283	2.170	
1	4	10	2	0.443	2567	3.010	
0	5	0	4	0	5134	5124	

FH*Vb*: Hexane fraction of *Vernonanthura brasiliana*; Anf-B: Amphotericin-B; IC_50_: inhibitory concentration for 50% of parasites. FIC_50_: fractional inhibitory concentrations; ∑FIC_50_: sum of fractional inhibitory concentrations; FICI: fractional inhibitory concentration index.

**Table 5:** Cytotoxic evaluation of crude extract and fractions from leaves of *Vernonanthura brasiliana* (L.) H. Rob, after 48 h of treatment in RAW 264.7 cells.

Natural Compounds	CC_50_ (µg·mL^−1^)	95% CI (µg·mL^−1^)	R²	SI
EB*Vb*	<8	ND	ND	ND
FH*Vb*	314.8	90.23–106.6	0.964	25.30
FAE*Vb*	32.4	95.60–147.4	0.979	1, 14
DMSO (40µg·mL^−1^)	<1	ND	ND	ND

CC_50_: cytotoxic concentration of 50% of cells. CI: confidence interval. R²: correlation of the data. SI: selectivity index.

## Data Availability

The data presented in this study are available in article.

## References

[1] Mashayekhi-Ghoyonlo V., Kiafar B., Rohani M., Esmaeili H., Erfanian-Taghvaee M.R. (2015). Correlation between Socioeconomic Status and Clinical Course in Patients with Cutaneous Leishmaniasis. J. Cutan. Med. Surg..

[2] Alvar J., Vélez I.D., Bern C., Herrero M., Desjeux P., Cano J., Jannin J., den Boer M. (2012). Who Leishmaniasis Control the WHO Leishmaniasis Control Team Leishmaniasis Worldwide and Global Estimates of Its Incidence. PLoS ONE.

[3] Jennings Y.L., de Souza A.A.A., Ishikawa E.A., Shaw J., Lainson R., Silveira F. (2014). Phenotypic characterization of*Leishmania* spp. causing cutaneous leishmaniasis in the lower Amazon region, western Pará state, Brazil, reveals a putative hybrid parasite,*Leishmania*(*Viannia*)*guyanensis* × *Leishmania*(*Viannia*)*shawi shawi*. Parasite.

[4] Silveira F.T., Lainson R., Corbett C.E.P. (2004). Clinical and immunopathological spectrum of American cutaneous leishmaniasis with special reference to the disease in Amazonian Brazil: A review. Mem. Inst. Oswaldo Cruz..

[5] Cock I., Selesho M., Van Vuuren S. (2018). A review of the traditional use of southern African medicinal plants for the treatment of selected parasite infections affecting humans. J. Ethnopharmacol..

[6] Rocha L., Almeida J., Macêdo R., Barbosa-Filho J. (2005). A review of natural products with antileishmanial activity. Phytomedicine.

[7] Singh N., Mishra B.B., Bajpai S., Singh R.K., Tiwari V.K. (2013). Natural product based leads to fight against leishmaniasis. Bioorg. Med. Chem..

[8] Wang Z., Yang L. (2021). Chinese herbal medicine: Fighting SARS-CoV-2 infection on all fronts. J. Ethnopharmacol..

[9] Abdallah H., El-Halawany A., Sirwi A., El-Araby A., Mohamed G., Ibrahim S., Koshak A., Asfour H., Awan Z., Elfaky M.A. (2021). Repurposing of Some Natural Product Isolates as SARS-CoV-2 Main Protease Inhibitors via In Vitro Cell Free and Cell-Based Antiviral Assessments and Molecular Modeling Approaches. Pharmaceuticals.

[10] Huo J.-L., Fu W.-J., Liu Z.-H., Lu N., Jia X.-Q., Liu Z.-S. (2022). Research advance of natural products in tumor immunotherapy. Front. Immunol..

[11] Yang L., Wang Z. (2021). Natural Products, Alone or in Combination with FDA-Approved Drugs, to Treat COVID-19 and Lung Cancer. Biomedicines.

[12] Neto R.N.M., Setúbal R.F.B., Higino T.M.M., Castro M.C., Da Silva L.C.N., Aliança A.S.D.S. (2019). Asteraceae Plants as Sources of Compounds Against Leishmaniasis and Chagas Disease. Front. Pharmacol..

[13] Hameed H., King E., Doleckova K., Bartholomew B., Hollinshead J., Mbye H., Ullah I., Walker K., Van Veelen M., Abou-Akkada S. (2021). Temperate Zone Plant Natural Products—A Novel Resource for Activity against Tropical Parasitic Diseases. Pharmaceuticals.

[14] de Sousa D.F., de Araújo M.F.M., de Mello V.D., Damasceno M.M.C., de Freitas R.W.J.F. (2020). Cost-Effectiveness of Passion Fruit Albedo versus Turmeric in the Glycemic and Lipaemic Control of People with Type 2 Diabetes: Randomized Clinical Trial. J. Am. Coll. Nutr..

[15] Sereno A.B., Pinto C.D., Andrade F.A., da Silva M.A.B., Garcia A.C., Krüger C.C.H., Reason I.J.D.M. (2022). Effects of okra (*Abelmoschus esculentus* (L.) Moench) on glycemic markers in animal models of diabetes: A systematic review. J. Ethnopharmacol..

[16] Li S.-Z., Zeng S.-L., Liu E.-H. (2021). Anti-obesity natural products and gut microbiota. Food Res. Int..

[17] de Almeida A.M., Fonseca C.R., Prado P.I., Almeida-Neto M., Diniz S., Kubota U., Braun M.R., Raimundo R.L.G., dos Anjos L.A., Mendonça T.G. (2005). Diversidade e ocorrência de Asteraceae em cerrados de São Paulo. Biota Neotrop..

[18] De Mesquita M.L., Desrivot J., Bories C., Fournet A., De Paula J.E., Grellier P., Espindola L. (2005). Antileishmanial and trypanocidal activity of Brazilian Cerrado plants. Mem. Inst. Oswaldo Cruz..

[19] Nishimuta H., Rossi A., Yamashita O., Pena G., Santos P., Giustina L., Rossi F. (2019). Leaf and Root Allelopathic Potential of the Vernonanthura brasiliana. Planta Daninha.

[20] Toyang N.J., Verpoorte R. (2013). A review of the medicinal potentials of plants of the genus Vernonia (Asteraceae). J. Ethnopharmacol..

[21] Adedapo A.A., Aremu O.J., Oyagbemi A. (2014). Anti-oxidant, anti-inflammatory and antinociceptive properties of the acetone leaf extract of vernonia amygdalina in some laboratory animals. Adv. Pharm. Bull..

[22] Cáceres A.L., Flores-Giubi M.E., Romero-Rodríguez M.C., Alvarenga N.L. (2017). In vitro anthelmintic activity and chemical composition of methanol extracts and fractions of Croton paraguayensis and Vernonia brasiliana against Eisenia fetida. Asian Pac. J. Trop. Dis..

[23] de Arias A.R., Ferro E., Inchausti A., Ascurra M., Acosta N., Rodriguez E., Fournet A. (1995). Mutagenicity, insecticidal and trypanocidal activity of some Paraguayan Asteraceae. J. Ethnopharmacol..

[24] Rocha M.F.G., De Aguiar F.L.N., Brilhante R.S.N., Cordeiro R.D.A., Teixeira C.E.C., Castelo-Branco D.D.S.C.M., Paiva M.D.A.N., Zeferino J.P.O., Mafezoli J., Sampaio C.M.D.S. (2011). Extratos de Moringa oleifera e *Vernonia* sp. sobre Candida albicans e Microsporum canis isolados de cães e gatos e análise da toxicidade em *Artemia* sp.. Ciência Rural.

[25] da Silva V.D., Almeida-Souza F., Teles A.M., Neto P.A., Mondego-Oliveira R., Filho N.E.M., Taniwaki N.N., Abreu-Silva A.L., Calabrese K.D.S., Filho V.E.M. (2018). Chemical composition of Ocimum canum Sims. essential oil and the antimicrobial, antiprotozoal and ultrastructural alterations it induces in Leishmania amazonensis promastigotes. Ind. Crop. Prod..

[26] Maia A.I.V., Torres M.C.M., Pessoa O.D.L., De Menezes J.E.S.A., Costa S.M.O., Nogueira V.L.R., Melo V.M.M., De Souza E.B., Cavalcante M.G.B., Albuquerque M.R.J.R. (2010). Óleos essenciais das folhas de Vernonia Remotiflora e Vernonia Brasiliana: Composição química e atividade biológica. Quim. Nova.

[27] Abreu P.M., Martins E.S., Kayser O., Bindseil K.-U., Siems K., Seemann A., Frevert J. (1999). Antimicrobial, antitumor and antileishmania screening of medicinal plants from Guinea-Bissau. Phytomedicine.

[28] Mondêgo-Oliveira R., Sousa J.C.D.S., Moragas-Tellis C.J., de Souza P.V.R., Chagas M.D.S.D.S., Behrens M.D., Hardoim D.D.J., Taniwaki N.N., Chometon T.Q., Bertho A.L. (2021). *Vernonia brasiliana* (L.) Druce induces ultrastructural changes and apoptosis-like death of Leishmania infantum promastigotes. Biomed. Pharmacother..

[29] Dennington R., Keith T.A., Millam J.M. (2016). GaussView5. https://www.scirp.org/(S(vtj3fa45qm1ean45vvffcz55))/reference/ReferencesPapers.aspx?ReferenceID=1958990.

[30] Frisch M.J., Trucks G.W., Schlegel H.B., Scuseria G.E., Robb M.A., Cheeseman J.R., Scalmani G., Barone V., Petersson G.A., Nakatsuji H. (2016). Gaussian 09. https://www.scirp.org/(S(351jmbntvnsjt1aadkozje))/reference/referencespapers.aspx?referenceid=1989943.

[31] Trott O., Olson A.J. (2010). AutoDock Vina: Improving the speed and accuracy of docking with a new scoring function, efficient optimization, and multithreading. J. Comput. Chem..

[32] Morris G.M., Huey R., Lindstrom W., Sanner M.F., Belew R.K., Goodsell D.S., Olson A.J. (2009). AutoDock4 and AutoDockTools4: Automated docking with selective receptor flexibility. J. Comput. Chem..

[33] Lopes A.J.O., Calado G.P., Fróes Y.N., de Araújo S.A., França L.M., Paes A.M.D.A., de Morais S.V., da Rocha C.Q., Vasconcelos C.C. (2022). Plant Metabolites as SARS-CoV-2 Inhibitors Candidates: In Silico and In Vitro Studies. Pharmaceuticals.

[34] Riss T.L., Moravec R.A., Niles A.L., Duellman S., Benink H.A., Worzella T.J., Minor L. (2016). Cell Viability Assays. Assay Guid. Man. https://www.ncbi.nlm.nih.gov/books/NBK144065/.

[35] Fivelman Q.L., Adagu I.S., Warhurst D.C. (2004). Modified Fixed-Ratio Isobologram Method for Studying In Vitro Interactions between Atovaquone and Proguanil or Dihydroartemisinin against Drug-Resistant Strains of *Plasmodium falciparum*. Antimicrob. Agents Chemother..

[36] Mosmann T. (1983). Rapid colorimetric assay for cellular growth and survival: Application to proliferation and cytotoxicity assays. J. Immunol. Methods.

[37] Colasso A.H.M., Barros T.F., Figueiredo I.F.D.S., Junior A.R.C., Fernandes E.S., Uchoa M.R.B., da Silva L.C.N. (2019). The latex of *Euphorbia tirucalli* inhibits staphyloxanthin production and protects *Tenebrio molitor* larvae against *Staphylococcus aureus* infection. Nat. Prod. Res..

[38] Gobbo-Neto L., Lopes N.P. (2007). Plantas Medicinais: Fatores de Influência No Conteúdo de Metabólitos Secundários. Quim. Nova.

[39] Fugita J.M.S., Pereira T.B.C., Banzato T.C., Kitajima E.W., Souto E.R., Bedendo I.P. (2017). Two distinct 16SrIII phytoplasma subgroups are associated with shoot proliferation in Vernonia brasiliana, a wild species inhabiting the Brazilian savanna. Trop. Plant Pathol..

[40] Tuck S., Patel H., Safi E., Robinson C. (1991). Lanosterol 14 alpha-demethylase (P45014DM): Effects of P45014DM inhibitors on sterol biosynthesis downstream of lanosterol. J. Lipid Res..

[41] Sen R., Chatterjee M. (2011). Plant derived therapeutics for the treatment of Leishmaniasis. Phytomedicine.

[42] Santos G.C.d.O., Vasconcelos C.C., Lopes A.J.O., do S. (2018). de Sousa Cartágenes, M.; Filho, A.K.D.B.; do Nascimento, F.R.F.; Ramos, R.M.; Pires, E.R.R.B.; de Andrade, M.S.; Rocha, F.M.G.; et al. Candida Infections and Therapeutic Strategies: Mechanisms of Action for Traditional and Alternative Agents. Front. Microbiol..

[43] Hargrove T.Y., Friggeri L., Wawrzak Z., Qi A., Hoekstra W.J., Schotzinger R.J., York J.D., Guengerich F.P., Lepesheva G.I. (2017). Structural analyses of Candida albicans sterol 14α-demethylase complexed with azole drugs address the molecular basis of azole-mediated inhibition of fungal sterol biosynthesis. J. Biol. Chem..

[44] Taran M., Mohebali M., Esmaeli J. (2010). In Vivo Efficacy of Gum Obtained Pistacia Atlantica in Experimental Treatment of Cutaneous Leishmaniasis. Iran. J. Public Heal..

[45] Ahmed Z.B., Yousfi M., Viaene J., Dejaegher B., Demeyer K., Heyden Y. (2021). Vander Four Pistacia Atlantica Subspecies (Atlantica, Cabulica, Kurdica and Mutica): A Review of Their Botany, Ethnobotany, Phytochemistry and Pharmacology. J. Ethnopharmacol..

[46] Blainski A., Gionco B., Oliveira A.G., Andrade G., Scarminio I.S., Silva D.B., Lopes N.P., Mello J.C. (2017). Antibacterial activity of Limonium brasiliense (Baicuru) against multidrug-resistant bacteria using a statistical mixture design. J. Ethnopharmacol..

[47] Salem M.M., Werbovetz K.A. (2004). Antiprotozoal Compounds from Psorothamnus polydenius. J. Nat. Prod..

[48] Sandjo L.P., de Moraes M.H., Kuete V., Kamdoum B.C., Ngadjui B.T., Steindel M. (2016). Individual and combined antiparasitic effect of six plant metabolites against Leishmania amazonensis and Trypanosoma cruzi. Bioorg. Med. Chem. Lett..

[49] DAS B.B., Sen N., Roy A., Dasgupta S.B., Ganguly A., Mohanta B.C., Dinda B., Majumder H.K. (2006). Differential induction of Leishmania donovani bi-subunit topoisomerase I-DNA cleavage complex by selected flavones and camptothecin: Activity of flavones against camptothecin-resistant topoisomerase I. Nucleic Acids Res..

[50] Mittra B., Saha A., Chowdhury A.R., Pal C., Mandal S., Mukhopadhyay S., Bandyopadhyay S., Majumder H.K. (2000). Luteolin, an Abundant Dietary Component is a Potent Anti-leishmanial Agent that Acts by Inducing Topoisomerase II-mediated Kinetoplast DNA Cleavage Leading to Apoptosis. Mol. Med..

[51] Manjolin L.C., dos Reis M.B.G., Maquiaveli C.D.C., Santos-Filho O.A., da Silva E.R. (2013). Dietary flavonoids fisetin, luteolin and their derived compounds inhibit arginase, a central enzyme in Leishmania (Leishmania) amazonensis infection. Food Chem..

[52] Albayrak S., Aksoy A., Sağdiç O., Budak Ü. (2010). Phenolic compounds and antioxidant and antimicrobial properties of Helichrysum species collected from eastern Anatolia, Turkey. Turk. J. Biol..

[53] Morikawa T., Ninomiya K., Akaki J., Kakihara N., Kuramoto H., Matsumoto Y., Hayakawa T., Muraoka O., Wang L.-B., Wu L.-J. (2015). Dipeptidyl peptidase-IV inhibitory activity of dimeric dihydrochalcone glycosides from flowers of Helichrysum arenarium. J. Nat. Med..

[54] Tabatabaei S.M., Farimani M.M., Nejad-Ebrahimi S., Salehi P. (2020). Phytochemical study of Tanacetum sonbolii aerial parts and the antiprotozoal activity of its components. Biointerface Res. Appl. Chem..

[55] Czinner E., Hagymási K., Blázovics A., Kéry A., Szőke E., Lemberkovics E. (2000). In vitro antioxidant properties of *Helichrysum arenarium* (L.) Moench. J. Ethnopharmacol..

[56] Kefi S., Essid R., Mkadmini K., Kefi A., Haddada F.M., Tabbene O., Limam F. (2018). Phytochemical investigation and biological activities of *Echium arenarium* (Guss) extracts. Microb. Pathog..

[57] de Oliveira D.P., de Almeida L., Marques M.J., de Carvalho R.R., Dias A.L.T., da Silva G.A., de Pádua R.M., Braga F.C., da Silva M.A. (2019). Exploring the bioactivity potential of *Leonotis nepetifolia*: Phytochemical composition, antimicrobial and antileishmanial activities of extracts from different anatomical parts. Nat. Prod. Res..

[58] Fonseca-Silva F., Canto-Cavalheiro M.M., Menna-Barreto R.F.S., Almeida-Amaral E.E. (2015). Effect of Apigenin on *Leishmania amazonensis* Is Associated with Reactive Oxygen Species Production Followed by Mitochondrial Dysfunction. J. Nat. Prod..

[59] Naddaf N., Haddad S. (2020). Apigenin effect against *Leishmania* tropica amastigotes in vitro. J. Parasit. Dis..

[60] Cruz E.D.M., da Silva E.R., Maquiaveli C.D.C., Alves E.S.S., Lucon J.F., dos Reis M.B.G., de Toledo C.E.M., Cruz F.G., Vannier-Santos M.A. (2013). Leishmanicidal activity of Cecropia pachystachya flavonoids: Arginase inhibition and altered mitochondrial DNA arrangement. Phytochemistry.

[61] Emiliano Y.S.S., Almeida-Amaral E.E. (2018). Efficacy of Apigenin and Miltefosine Combination Therapy against Experimental Cutaneous Leishmaniasis. J. Nat. Prod..

[62] Gonçalves-Oliveira L.F., Souza-Silva F., Côrtes L.M.D.C., Veloso L.B., Pereira B.A.S., Cysne-Finkelstein L., Lechuga G.C., Bourguignon S.C., Almeida-Souza F., Calabrese K.D.S. (2019). The combination therapy of meglumine antimoniate and oxiranes (epoxy-α-lapachone and epoxymethyl-lawsone) enhance the leishmanicidal effect in mice infected by *Leishmania* (*Leishmania*) amazonensis. Int. J. Parasitol. Drugs Drug Resist..

[63] Pastor J., García M., Steinbauer S., Setzer W.N., Scull R., Gille L., Monzote L. (2015). Combinations of ascaridole, carvacrol, and caryophyllene oxide against *Leishmania*. Acta Trop..

[64] dos Santos D.B., Lemos J.A., Miranda S.E.M., Di Filippo L.D., Duarte J.L., Ferreira L.A.M., Barros A.L.B., Oliveira A.E.M.F.M. (2022). Current Applications of Plant-Based Drug Delivery Nano Systems for Leishmaniasis Treatment. Pharmaceutics.

[65] Mehrizi T.Z., Khamesipour A., Ardestani M.S., Shahmabadi H.E., Hoseini M.H.M., Mosaffa N., Ramezani A. (2019). Comparative analysis between four model nanoformulations of amphotericin B-chitosan, amphotericin B-dendrimer, betulinic acid-chitosan and betulinic acid-dendrimer for treatment of *Leishmania* major: Real-time PCR assay plus. Int. J. Nanomed..

[66] Mehrizi T.Z., Ardestani M.S., Hoseini M.H.M., Khamesipour A., Mosaffa N., Ramezani A. (2018). Novel Nanosized Chitosan-Betulinic Acid Against Resistant *Leishmania* Major and First Clinical Observation of such parasite in Kidney. Sci. Rep..

